# A population-based cohort study on the risk of obstructive lung disease after bilateral oophorectomy

**DOI:** 10.1038/s41533-022-00317-4

**Published:** 2022-11-15

**Authors:** Trinh T. Nguyen, Carin Y. Smith, Liliana Gazzuola Rocca, Walter A. Rocca, Robert Vassallo, Megan M. Dulohery Scrodin

**Affiliations:** 1 Department of Pulmonary & Critical Care Medicine, Mayo Clinic, Rochester, MN USA; 2 Division of Clinical Trials and Biostatistics, Department of Quantitative Health Sciences, Mayo Clinic, Rochester, MN USA; 3 Division of Epidemiology, Department of Quantitative Health Sciences, Mayo Clinic, Rochester, MN USA; 4 Women’s Health Research Center, Mayo Clinic, Rochester, MN USA; 5 Department of Neurology, Mayo Clinic, Rochester, MN USA

**Keywords:** Chronic obstructive pulmonary disease, Asthma

## Abstract

There is increasing evidence that sex hormones may impact the development of obstructive lung disease (OLD). Therefore, we studied the effect of bilateral oophorectomy (oophorectomy) on the development of OLD. Women were identified from the Mayo Clinic Cohort Study of Oophorectomy and Aging-2. Data were collected using the Rochester Epidemiology Project records-linkage system. A total of 1653 women who underwent oophorectomy and 1653 referent women of similar age were assessed for OLD using diagnostic codes and medical record abstraction. Women who underwent oophorectomy had an overall higher risk of all OLD, all chronic obstructive pulmonary disease (COPD), emphysema, and chronic bronchitis but not of all asthma, confirmed asthma, or confirmed COPD. The association with all OLD was stronger in women who were age ≤45 years at oophorectomy, never smokers, non-obese, and in women with benign indications; however, the interactions were not statistically significant. There was an increased risk of all asthma in women age ≤45 years at oophorectomy who took estrogen therapy. Never smokers of all ages had a stronger association of oophorectomy with all asthma and all COPD, whereas smokers had a stronger association of oophorectomy with emphysema and chronic bronchitis. Non-obese women of all ages had a stronger association of oophorectomy with all COPD, emphysema, and chronic bronchitis. The results of this study combined with the increased risk of several chronic diseases reported in previous studies suggest that oophorectomy in premenopausal women should be avoided unless there is clear evidence of a high genetic risk of ovarian cancer.

## Introduction

It is now apparent that some diseases affect women disproportionately more than men. Although behavioral and environmental factors play important roles in the development of obstructive lung disease (OLD), the potential role of sex-specific factors is only partially understood^1–5^.

There is abundant evidence implicating sex hormones in the pathophysiology of asthma in women. Asthma has a bimodal age distribution, affecting males predominately in childhood and women predominately after puberty. Prevalence of severe asthma and healthcare utilization rises in women as age increases until reaching menopause^6^. Among women with asthma, the forced expiratory volume at 1 s (FEV_1_) and diffusing capacity for carbon monoxide vary during the menstrual cycle. Lung function dips in the luteal phase when there is a peak in both estrogen and progesterone^7,8^. In this premenstrual phase, one-third of women with asthma also experience worsening of their respiratory symptoms that appear to be partially reversed by contraceptive use^9,10^. Furthermore, early menarche has been associated with increased risk of asthma^11^.

In addition, there are sex differences in chronic obstructive pulmonary disease (COPD). The National Health Interview Survey described a higher self-reported prevalence of COPD in women than in men^12^. In addition, the National Health and Nutrition Examination Survey (NHANES) I and III studies further demonstrated that the mortality associated with COPD is higher in men but is declining over time more rapidly in men, narrowing the mortality gap between sexes^13^. Changes in smoking trends may influence these differences, but the NHANES III study also demonstrated that 80% of non-smokers with COPD were women^14^. Among smokers, women have increased susceptibility with more rapid FEV_1_ and computed tomography (CT) density decline than their male counterparts^15–17^. In addition, women have higher reported disease burden, depression, and worse quality of life^18^.

Female reproductive hormones, namely estrogen and progesterone, influence lung function as women advance in their reproductive lifespan. Estradiol is the major active estrogen form in humans, and binds to estrogen receptors that are broadly distributed in many cell types in the lung^5^. Activation of these receptors can modulate immune cells and airway structural functions in complex ways.

Progesterone modulates airway function as well. Progesterone receptors are present on cilia of airway epithelial cells, and their activation decreases cilia beat frequency. Progesterone level is the highest in the luteal phase, when lung function has been found to be the lowest in menstruating women with asthma^7^.

Menopause marks the end of reproduction due to ovarian follicular depletion. Bilateral oophorectomy (oophorectomy) with or without concurrent hysterectomy may cause premature or early menopause which may lead to symptoms necessitating the use of hormonal replacement therapy, primarily estrogen therapy^19^. The effects of menopause on lung function and on predisposition to asthma and COPD are not well defined. Some studies have suggested that there is a decreased risk of asthma after spontaneous menopause, and reversal with hormone replacement therapy^20–23^. Furthermore, although some studies found that menopause was associated with higher FEV_1_, forced vital capacity (FVC), and FEV_1_/FVC^24^, other studies suggested that menopause was associated with worsened symptoms and worsened FEV_1_ and FVC^25,26^.

To further investigate the effects of sex hormones on lung pathology, we studied the effect of bilateral oophorectomy on the development of OLD using a population-based cohort study design.

## Methods

### Study population

Women were identified as part of the Mayo Clinic Cohort Study of Oophorectomy and Aging-2 (MOA-2)^27–29^, and data were collected using the Rochester Epidemiology Project medical records-linkage system. The system links outpatient and inpatient medical records from all medical care providers in Olmsted County, Minnesota^30–33^. The study included all premenopausal women who underwent bilateral oophorectomy or second unilateral oophorectomy for a nonmalignant indication before age 50 years (*n* = 1653), between 1 January 1988 and 31 December 2007 in Olmsted County, Minnesota. Simple random sampling was used to match each woman to a referent woman born in the same year (±1) who had not undergone bilateral oophorectomy or second unilateral oophorectomy before index date. For each pair, index date was the date of oophorectomy.

### Ethics

Women were not contacted directly as part of the study; therefore, written informed consent was not required. However, women were excluded if they did not authorize the use of their medical record for research (only 2.5% of women in the overall population)^33^. Research activities were approved by both the Mayo Clinic and the Olmsted Medical Center Review Boards.

### Assessment of characteristics at index date

Chronic conditions present at index date were electronically abstracted using the International Classification of Diseases diagnostic codes assigned to each woman before index date. We used the diagnostic codes recommended by the U.S. Department of Health and Human Services to define the following 15 chronic conditions: depression, substance abuse disorders, dementia, schizophrenia or psychosis, hyperlipidemia, hypertension, diabetes (both type 1 and type 2), cardiac arrhythmias, coronary artery disease, stroke, congestive heart failure, arthritis, cancer, osteoporosis, and chronic kidney disease^34,35^. Anxiety was added to the list because it was common in our population and was considered related to the aging process. By contrast, three additional chronic conditions were excluded from the list because they were uncommon in the study population (autism spectrum disorder, human immunodeficiency virus, and hepatitis). Finally, two additional chronic conditions were excluded because they were the outcomes of this study (asthma and COPD). In total, the study considered 16 chronic conditions. Each condition required two or more corresponding diagnostic codes separated by >30 days. Demographic information, clinical characteristics, surgical indication, and use of estrogen therapy were manually abstracted from the medical records in the Rochester Epidemiology Project by a nurse or by a physician (L.G.R.). Income at the census block group level was derived from U.S. Census data.

### Assessment of OLD and diagnostic criteria

The outcome of interest in this cohort study was OLD and included asthma and COPD. All diagnostic codes defined by the U.S. Department of Health and Human Services for asthma and COPD assigned at any time before or after index date through December 31, 2018 were obtained from the electronic indexes of the Rochester Epidemiology Project (Supplementary Table 1). To avoid false positives, subjects were required to meet specific screening criteria. We required one or more codes for asthma, COPD, or emphysema, and two or more codes separated by >90 days and <1 year for chronic bronchitis. Women who met the screening criteria before index date were considered as prevalent OLD and were excluded from all analyses. The medical records for all remaining women who met the screening criteria on or after index date were abstracted by a physician (T.T.N.) to confirm the presence of OLD and to determine specific type, date of onset, and other characteristics. We excluded women for whom medical record abstraction revealed that the date of onset was before index date (prevalent OLD).

All available pulmonary function tests (PFT) were independently reviewed by a physician (T.T.N.) and interpreted based on current guidelines. The Global Initiative for Chronic Obstructive Lung Disease (GOLD) 2019 guideline was used to define fixed obstruction for the diagnosis of confirmed COPD. The Global Initiative for Asthma (GINA) 2019 guideline was used to define variable obstruction for the diagnosis of confirmed asthma. Chest CT reports interpreted by a radiologist were additionally used to determine parenchymal abnormalities, including emphysema. We used these criteria to categorize the type of OLD (Table 1). Women could have more than one type of OLD to account for potential disease overlap^36^.

**Table 1:** Diagnostic criteria for specific types of obstructive lung disease (OLD).

OLD type and sub-type	Criteria
Asthma	
Confirmed	GINA 2019 definition - obstructive baseline spirometry (FEV_1_/FVC ratio <70%) from PFT with increase from baseline FEV_1_ post-bronchodilation (>0.2 L and >12%), and/or decrease in baseline FEV_1_ post-provocation (≥20% after methacholine or ≥15% after other types)
Probable	One or more diagnostic codes for asthma, and PFT without provocation study
Possible	One or more diagnostic codes for asthma without any PFT
COPD	
Confirmed	GOLD 2019 definition - obstructive spirometry post-bronchodilation (FEV_1_/FVC ratio <70%) from PFT
Probable	Non-specific pattern from PFT, with high airway resistance or high total lung capacity
Emphysema without airflow limitation	Findings of emphysema on chest CT, with non-obstructive findings from PFT
Emphysema without PFT	Findings of emphysema on chest CT without any PFT
Undifferentiated OLD	Obstructive baseline spirometry (FEV_1_/FVC ratio <70%) from PFT without variability evaluation
Chronic bronchitis	Two or more diagnostic codes for bronchitis separated by >90 days to ensure chronic state, but <1 year apart to avoid false positives
Other OLD	Non-specific pattern from PFT, without testing for small airway resistance or total lung capacity

*COPD* chronic obstructive pulmonary disease, *CT* computed tomography, *FEV*_*1*_ forced expiratory volume in 1 s, *FVC* forced vital capacity, *OLD* obstructive lung disease, *PFT* pulmonary function test.

We considered all types of OLD combined, including confirmed asthma, probable asthma, possible asthma, confirmed COPD, probable COPD, emphysema without airflow limitation, emphysema without PFT, undifferentiated OLD, chronic bronchitis, and other OLD. We also considered specific types for all asthma (confirmed, probable, and possible), confirmed asthma, all COPD (confirmed, probable, and emphysema), confirmed COPD, emphysema (without airflow limitation and without PFT), and chronic bronchitis (Table 1).

### Statistical analysis

Women were followed from their index date until the date of OLD (combined or specific type), death, last visit with a care provider in the Rochester Epidemiology Project, or the end of the study (December 31, 2018). The oophorectomy and referent cohorts were balanced at baseline using inverse probability weights derived from a logistic regression model including several characteristics at baseline: 16 chronic conditions (list provided above), years of education (≤12, 13–16, and >16), household income quartiles (<$42,000, $42,000–56,999, $57,000–71,999, ≥$72,000), race (white vs. nonwhite), body mass index (BMI; ≥ 30 kg/m^2^ vs. <30), cigarette smoking (current or former vs. never), age at index date (continuous), and calendar year (continuous). The inverse probability weights were calculated separately within each stratum to maximize the balance of the characteristics. After balancing using inverse probability weights, the standardized differences for all of the characteristics considered were below the recommended threshold of 0.10 (i.e., negligible imbalance between the two cohorts; data not shown).

Cox proportional hazards models were used to estimate hazard ratios (HRs) and 95% CIs with age as the time scale, and with each woman entering the risk set at her respective age at index date. The Cox models used robust sandwich covariance estimates to account for the inclusion of women in both cohorts (referent women who underwent subsequent bilateral oophorectomy) and for the use of estimated weights. The proportional hazards assumption was tested using graphical methods and adding time-dependent covariates to the models, and was met for all models. Cumulative incidence curves were estimated using the Kaplan-Meier method and included inverse probability weighting. Differences between the oophorectomy and referent cohorts were also measured using absolute risk increase (ARI) or reduction, calculated by subtracting the two absolute risks at 25 years after index date.

Analyses were performed for all women overall and separately within strata by age at oophorectomy (≤45 and 46–49 y), by estrogen therapy within each age group, by surgical indication, by smoking status (current or former vs. never), and by obesity (BMI ≥ 30 vs. <30 kg/m^2^). Stratification by age at oophorectomy, estrogen therapy, and surgical indication was based on the status of the woman who underwent oophorectomy in each pair. By contrast, stratification by smoking and obesity was based on the status for each woman separately (e.g., never smokers who underwent oophorectomy were compared with never smoker referent women). We also performed two sets of sensitivity analyses to (1) censor referent women at the time of bilateral oophorectomy if it was performed after index date, and (2) exclude women with any of the 16 chronic conditions at index date. Tests of statistical significance were conducted at the two-tailed alpha level of 0.05. Analyses were performed using SAS version 9.4 (SAS Institute Inc, Cary, NC).

### Reporting summary

Further information on research design is available in the Nature Research Reporting Summary linked to this article.

## Results

### Characteristics at index date

A total of 1653 women who underwent oophorectomy and 1653 referent women of similar age were assessed for OLD using diagnostic codes and medical record abstraction (Supplementary Fig. 1). A total of 317 (19.2%) women who underwent oophorectomy and 218 (13.2%) referent women had prevalent OLD before index date and were excluded from all analyses. In a case-control analysis at baseline, oophorectomy was associated with OLD before index date (odds ratio, 1.41; 95% CI, 1.15–1.73; *P* = 0.001; adjusted for the variables included in the inverse probability weights).

Table 2 shows the baseline characteristics for the 1336 women who underwent oophorectomy and 1435 referent women included in analyses for OLD outcomes. Women in the oophorectomy group were more frequently white, had higher BMI, and had more chronic conditions at index date. Most women (89.8%) underwent hysterectomy concurrent with oophorectomy.

**Table 2:** Sociodemographic and clinical characteristics at index date of women who underwent bilateral oophorectomy and referent women.

Characteristic	Bilateral oophorectomy (*n* = 1336)		Referent women (*n* = 1435)		*P* value
	N	%	N	%	
Age at index date (years)					0.68
≤45	825	61.8	897	62.5	
46–49	511	38.2	538	37.5	
Index year					0.22
1988–1997	628	47.0	641	44.7	
1998–2007	708	53.0	794	55.3	
Race					0.002
White	1300	97.3	1357	94.6	
Black	15	1.1	27	1.9	
Asian	18	1.3	46	3.2	
Other	3	0.2	5	0.3	
Hispanic ethnicity	16	1.2	22	1.5	0.45
Years of education^1^					0.27
≤12	409	30.7	420	30.0	
13–16	726	54.5	742	52.9	
>16	198	14.9	240	17.1	
Unknown	3		33		
Income quartiles^1^					0.35
<$42,000	317	23.8	353	24.6	
$42,000–56,999	357	26.8	358	25.0	
$57,000–71,999	348	26.2	356	24.8	
≥$72,000	308	23.2	367	25.6	
Unknown	6		1		
Body mass index (kg/m^2^)^1^					<0.001
<25.0	508	38.0	624	44.2	
25.0–29.9	390	29.2	421	29.8	
≥30.0	438	32.8	367	26.0	
Unknown	0		23		
Smoking status					0.24
Never	752	56.3	853	59.4	
Former	316	23.7	319	22.2	
Current	268	20.1	263	18.3	
Number of chronic conditions^2^					<0.001
0	702	52.5	925	64.5	
1	343	25.7	294	20.5	
2	166	12.4	140	9.8	
≥3	125	9.4	76	5.3	
Hysterectomy status					<0.001
None	15	1.1	1312	91.4	
Before	121	9.1	123	8.6	
Concurrent	1200	89.8	—	—	
Prior unilateral oophorectomy	120	9.0	43	3.0	<0.001
Indication for oophorectomy^3^					—
Benign ovarian condition	541	40.5	—	—	
No ovarian condition	795	59.5	—	—	

^1^Women with missing or unknown data were not included in the respective analysis. In the regression models used to derive inverse probability weights, women with unknown education were assigned to the ≤12 years group, women with unknown income were assigned to the $42,000–56,999 quartile, and women with unknown body mass index were assigned to the <30 kg/m^2^ group.

^2^A total of 16 chronic conditions defined by the US Department of Health and Human Services (DHHS), including depression, anxiety, substance abuse disorders, dementia, schizophrenia or psychosis, hyperlipidemia, hypertension, diabetes mellitus, cardiac arrhythmias, coronary artery disease, stroke, congestive heart failure, arthritis, cancer (all types), osteoporosis, and chronic kidney disease.

^3^The indication was listed by the gynecologist in the medical record at the time of oophorectomy.

### Follow-up and menopause characteristics

Supplementary Table 2 shows the follow-up characteristics and details about menopause in the oophorectomy and referent cohorts. The median length of follow-up was similar in the two cohorts. The median age at menopause was 44.0 years (interquartile range [IQR], 40.0–47.0) in women with oophorectomy and 50.0 years (IQR, 46.5–52.0) in referent women. Only 36% of the referent women who reached menopause received estrogen therapy.

Over a median of 18.6 years of follow-up (IQR 14.2–23.5), 214 women who underwent oophorectomy and 164 referent women developed *de novo* OLD (Supplementary Fig. 1). Supplementary Table 3 shows the clinical characteristics of women who developed any type of asthma (confirmed, probable, or possible) and any type of COPD (confirmed, probable, or emphysema). The age at asthma onset was similar between women who underwent oophorectomy (median, 51 y; IQR, 45–56) and referent women (median, 50 y; IQR, 45–56). However, women who underwent oophorectomy developed COPD at a younger age (median, 54 y; IQR, 52–59) compared with referent women (median, 60 y; IQR, 53–63). FEV_1_ and FVC from the earliest PFT with variable obstruction were similar for both groups of women with de novo asthma. Among women with *de novo* COPD, the FEV_1_ and FVC measurements were lower for referent women, possibly due to their older age at onset of COPD (Supplementary Table 3).

### Cohort study analyses

**Fig. 1:**
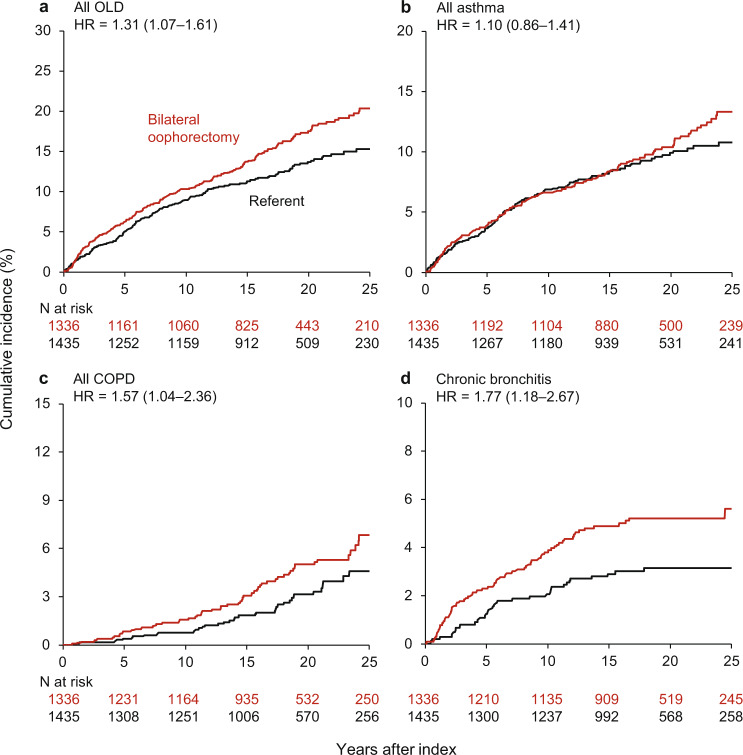
Cumulative incidence curves for obstructive lung disease, in all women who underwent bilateral oophorectomy compared with all referent women. **a** All OLD; **b**, all asthma; **c**, all COPD; **d**, chronic bronchitis. The curves were weighted using inverse probability weights derived from a logistic regression model including 16 chronic conditions present at index, years of education, quartiles of household income, race, body mass index, cigarette smoking, and age and calendar year at index date (details provided in text). *COPD* chronic obstructive pulmonary disease, *HR* hazard ratio, *OLD* obstructive lung disease.

The results for all OLD outcomes and for specific types of OLD after oophorectomy or index date are shown in Tables 3 and 4 and Supplementary Table 4. Women who underwent oophorectomy had an overall higher risk of all OLD (HR, 1.31; 95% CI, 1.07–1.61; ARI, 5.7%), all COPD (HR, 1.57; 95% CI, 1.04–2.36; ARI, 2.6%), emphysema (HR, 2.13; 95% CI, 1.18–3.85; ARI, 2.4%), and chronic bronchitis (HR, 1.77; 95% CI, 1.18–2.67; ARI, 2.8%) compared to referent women; but no significant difference in risk for all asthma, confirmed asthma, or confirmed COPD (Fig. 1). There was a stronger association of oophorectomy with all OLD among women aged ≤45 years at oophorectomy (overall and in those with estrogen therapy), women with benign ovarian indications, never smokers, and non-obese women. However, the interactions by age at oophorectomy, estrogen therapy, surgical indication, smoking status, and obesity were not statistically significant (Table 3 and Supplementary Fig. 2).

**Table 3:** Cumulative incidence of all obstructive lung disease, all asthma, and all chronic obstructive pulmonary disease overall and in strata by age at oophorectomy, estrogen therapy, surgical indication, cigarette smoking, and body mass index.

Strata	Bilateral oophorectomy				Referent women				Unweighted models^1^		Weighted models^2^	
	*N* at risk	Person-years	*N* of events	Absolute risk^3^ (95% CI)	*N* at risk	Person -years	*N* of events	Absolute risk^3^ (95% CI)	Hazard ratio (95% CI)	*P* value	Hazard ratio (95% CI)	*P* value
All OLD^4^	1336	21,747	214	21.0% (18.2–24.2)	1435	23,782	164	15.3% (13.1–17.8)	1.42 (1.17–1.74)	<0.001	1.31 (1.07–1.61)	0.01
Age ≤45 y	825	13,350	143	22.1% (18.6–26.1)	897	14,719	92	13.9% (11.4–17.0)	1.71 (1.32–2.21)	<0.001	1.53 (1.17–2.01)	0.002
Estrogen >49^5^	399	4876	40	15.0% (10.5–21.0)	378	4795	18	5.9% (3.6–9.7)	2.17 (1.25–3.79)	0.006	2.34 (1.32–4.16)	0.004
No estr. or ≤49	237	2196	18	11.6% (5.3–24.6)	238	2401	9	5.8% (2.7–12.4)	2.16 (0.97–4.81)	0.06	1.65 (0.72–3.76)	0.24
Age 46 to 49 y	511	8397	71	19.4% (14.9–25.0)	538	9063	72	17.4% (13.6–22.1)	1.06 (0.77–1.46)	0.74	1.04 (0.75–1.45)	0.80
Estrogen >49^5^	366	5758	48	20.4% (14.5–28.1)	374	6119	45	15.2% (11.1–20.7)	1.13 (0.76–1.69)	0.55	1.18 (0.78–1.78)	0.43
No estr. or ≤49	127	1625	14	13.0% (5.4–29.6)	125	1690	13	19.9% (9.2–40.1)	1.06 (0.50–2.23)	0.88	0.91 (0.41–2.01)	0.81
Benign indication^6^	541	8746	101	24.2% (19.8–29.4)	579	9608	68	15.4% (12.3–19.3)	1.61 (1.19–2.17)	0.002	1.47 (1.08–2.01)	0.01
No indication^7^	795	13,001	113	18.7% (15.2–22.7)	856	14,174	96	15.4% (12.5–18.9)	1.30 (0.99–1.70)	0.06	1.18 (0.89–1.57)	0.24
Ever smokers	584	9088	121	27.8% (23.0–33.3)	582	9482	101	22.5% (18.6–27.1)	1.23 (0.95–1.58)	0.11	1.22 (0.94–1.58)	0.13
Never smokers	752	12,659	93	16.5% (13.3–20.3)	853	14,300	63	9.5% (7.4–12.2)	1.73 (1.25–2.38)	<0.001	1.52 (1.09–2.13)	0.01
BMI ≥ 30 kg/m^2^	438	6593	89	28.2% (22.4–35.2)	367	5723	66	22.4% (17.3–28.7)	1.18 (0.86–1.61)	0.31	1.17 (0.85–1.61)	0.35
BMI < 30 kg/m^2^	898	15,154	125	18.2% (15.1–21.8)	1068	18,059	98	12.5% (10.2–15.2)	1.51 (1.16–1.96)	0.002	1.42 (1.09–1.85)	0.01
All asthma	1336	22,842	133	13.3% (11.1–16.0)	1435	24,276	121	10.8% (9.0–12.9)	1.17 (0.92–1.49)	0.21	1.10 (0.86–1.41)	0.43
Age ≤45 y	825	14,147	89	14.4% (11.5–17.9)	897	14,984	68	9.7% (7.6–12.3)	1.39 (1.02–1.89)	0.04	1.33 (0.96–1.83)	0.09
Estrogen >49^5^	416	5270	26	10.2% (6.5–15.8)	387	4971	9	3.0% (1.4–6.5)	2.73 (1.28–5.82)	0.01	3.26 (1.50–7.10)	0.003
No estr. or ≤49	249	2384	11	8.4% (3.1–21.7)	239	2415	7	2.7% (1.1–6.4)	1.58 (0.61–4.09)	0.34	1.49 (0.53–4.24)	0.45
Age 46 to 49 y	511	8695	44	11.4% (8.3–15.6)	538	9292	53	12.2% (9.1–16.2)	0.89 (0.60–1.31)	0.54	0.87 (0.58–1.29)	0.48
Estrogen >49^5^	368	6034	27	10.5% (7.0–15.6)	375	6296	31	10.2% (7.0–14.7)	0.91 (0.55–1.51)	0.72	0.91 (0.54–1.53)	0.72
No estr. or ≤49	127	1646	10	11.5% (4.3–29.0)	125	1741	9	9.8% (4.4–20.9)	1.17 (0.48–2.85)	0.73	1.09 (0.43–2.77)	0.86
Benign indication^6^	541	9309	60	15.0% (11.5–19.5)	579	9832	48	10.3% (7.8–13.7)	1.30 (0.90–1.90)	0.17	1.25 (0.85–1.84)	0.26
No indication^7^	795	13,532	73	11.9% (9.3–15.3)	856	14,444	73	11.2% (8.7–14.2)	1.09 (0.79–1.51)	0.61	1.02 (0.73–1.42)	0.92
Ever smokers	584	9866	59	12.8% (9.8–16.8)	582	9824	68	14.5% (11.4–18.3)	0.84 (0.60–1.18)	0.31	0.85 (0.60–1.19)	0.33
Never smokers	752	12,975	74	13.8% (10.8–17.6)	853	14,452	53	7.8% (5.9–10.3)	1.60 (1.12–2.27)	0.009	1.47 (1.01–2.12)	0.04
BMI ≥ 30 kg/m^2^	438	6999	61	19.0% (14.4–24.7)	367	5949	50	15.8% (11.8–20.8)	1.04 (0.72–1.50)	0.82	1.03 (0.71–1.51)	0.86
BMI < 30 kg/m^2^	898	15,843	72	11.0% (8.6–14.1)	1068	18,327	71	8.6% (6.8–11.0)	1.16 (0.84–1.61)	0.36	1.16 (0.83–1.60)	0.39
All COPD^8^	1336	23,909	59	7.2% (5.3–9.6)	1435	25,494	37	4.6% (3.2–6.4)	1.69 (1.13–2.53)	0.01	1.57 (1.04–2.36)	0.03
Age ≤45 y	825	14,860	40	8.1% (5.7–11.5)	897	15,699	20	3.7% (2.3–5.8)	2.10 (1.23–3.59)	0.007	1.88 (1.09–3.25)	0.02
Estrogen >49^5^	438	5544	21	7.3% (4.2–12.5)	403	5191	8	3.2% (1.5–6.6)	2.45 (1.09–5.53)	0.03	2.21 (0.92–5.30)	0.07
No estr. or ≤49	266	2547	13	4.5% (1.7–11.5)	251	2579	5	3.4% (1.1–10.3)	2.63 (0.94–7.37)	0.07	1.52 (0.45–5.19)	0.50
Age 46 to 49 y	511	9050	19	5.2% (3.1–8.8)	538	9795	17	6.0% (3.5–10.1)	1.20 (0.62–2.30)	0.59	1.25 (0.64–2.46)	0.52
Estrogen >49^5^	374	6289	14	5.1% (2.8–9.0)	381	6675	12	6.4% (3.2–12.3)	1.23 (0.57–2.66)	0.60	1.30 (0.56–2.98)	0.54
No estr. or ≤49	127	1737	4	2.1% (0.6–7.7)	127	1842	4	10.7% (3.4–31.0)	1.06 (0.28–3.99)	0.94	0.58 (0.14–2.33)	0.44
Benign indication^6^	541	9710	32	9.4% (6.4–13.6)	579	10,173	19	5.2% (3.3–8.3)	1.77 (1.02–3.08)	0.04	1.65 (0.94–2.89)	0.08
No indication^7^	795	14,199	27	5.7% (3.7–8.9)	856	15,321	18	4.1% (2.5–6.8)	1.60 (0.89–2.87)	0.11	1.52 (0.82–2.79)	0.18
Ever smokers	584	10,151	51	13.9% (10.2–18.8)	582	10,361	36	9.6% (6.7–13.6)	1.48 (0.98–2.24)	0.06	1.45 (0.95–2.21)	0.08
Never smokers	752	13,758	8	2.0% (0.9–4.2)	853	15,134	1	0.1% (0.0–0.9)	8.53 (1.09–66.6)	0.04	10.4 (1.28–84.6)	0.03
BMI ≥ 30 kg/m^2^	438	7559	17	6.5% (3.6, 11.6)	367	6493	14	6.7% (3.5, 12.5)	1.03 (0.53–2.00)	0.93	1.19 (0.60–2.35)	0.62
BMI < 30 kg/m^2^	898	16,350	42	7.4% (5.3, 10.4)	1068	19,002	23	3.8% (2.4, 5.8)	2.13 (1.28–3.54)	0.003	1.94 (1.16–3.24)	0.01

*BMI* body mass index, *CI* confidence interval, *COPD* chronic obstructive pulmonary disease, *OLD* obstructive lung disease.

^1^Hazard ratios were calculated using Cox proportional hazards models with age as the time scale.

^2^Hazard ratios were calculated using Cox proportional hazards models with age as the time scale and including inverse probability weights derived from a logistic regression model including 16 chronic conditions present at baseline, years of education (≤12, 13–16, >16), quartiles of household income (<$42,000, $42,000–56,999, $57,000–71,999, ≥$72,000), race (white vs. nonwhite), BMI (≥30 kg/m^2^ vs. <30), cigarette smoking (current or former vs. never), age at index date (continuous), and calendar year at index date (continuous). These weights were calculated separately in each stratum to maximize the balance at index date. A significant interaction was found by smoking for all asthma (*P* = 0.03). No significant interactions were found by age, estrogen therapy, indication, or by BMI for any of the three outcomes.

^3^Absolute cumulative risk at 25 years after bilateral oophorectomy (or index date) calculated using the Kaplan-Meier method and including inverse probability weights derived from a logistic regression model. These weights were calculated in each stratum to maximize the balance at index date.

^4^Includes all asthma (confirmed, probable, and possible), all COPD (confirmed, probable, and emphysema), undifferentiated OLD, other OLD, and chronic bronchitis.

^5^Women who were taking systemic estrogen therapy (only oral or transdermal) on their 50th birth date, after bilateral oophorectomy. Women who died or were lost to follow-up prior to their 50th birth date, or had not reached age 50 years as of 31 December 2018 were not included in this analysis. Follow-up for these analyses was started at age 50 years.

^6^The benign condition (e.g., cysts, endometrioma) was listed by the gynecologist in the medical record at the time of oophorectomy, but may not have been the sole indication for the surgery.

^7^Women without a benign ovarian condition. Historically, the terms “prophylactic”, “elective”, or “incidental” oophorectomy were used; however, we prefer to avoid these terms.

^8^Includes confirmed COPD, probable COPD, and emphysema (without airflow limitation and without pulmonary function testing).

**Table 4:** Cumulative incidence of emphysema and chronic bronchitis overall and in strata by age at oophorectomy, estrogen therapy, surgical indication, cigarette smoking, and body mass index.

Strata	Bilateral oophorectomy				Referent women				Unweighted models^1^		Weighted models^2^	
	*N* at risk	Person-years	*N* of events	Absolute risk^3^ (95% CI)	*N* at risk	Person -years	*N* of events	Absolute risk^3^ (95% CI)	Hazard ratio (95% CI)	*P* value	Hazard ratio (95% CI)	*P* value
Emphysema	1336	24,204	33	4.2% (2.8–6.3)	1435	25,680	15	1.8% (1.1–3.2)	2.32 (1.29–4.15)	0.005	2.13 (1.18–3.85)	0.01
Age ≤45 y	825	15,031	22	4.7% (2.9–7.6)	897	15,798	10	2.3% (1.2–4.2)	2.28 (1.08–4.82)	0.03	1.77 (0.83–3.79)	0.14
Estrogen >49^4^	439	5660	11	3.8% (1.7–8.4)	405	5257	4	1.8% (0.6–4.9)	2.54 (0.81–7.96)	0.11	1.86 (0.55–6.26)	0.32
No estr. or ≤49	268	2591	8	3.4% (1.1–10.5)	252	2599	3	3.1% (0.9–10.2)	2.67 (0.71–10.0)	0.15	1.35 (0.33–5.47)	0.68
Age 46 to 49 y	511	9173	11	3.0% (1.4–6.5)	538	9883	5	1.5% (0.6–4.1)	2.33 (0.82–6.67)	0.11	2.56 (0.87–7.54)	0.09
Estrogen >49^4^	374	6395	7	2.6% (1.0–6.3)	381	6737	4	1.9% (0.6–6.1)	1.84 (0.54–6.26)	0.33	1.96 (0.54–7.12)	0.31
No estr. or ≤49	128	1751	4	2.1% (0.6–7.7)	127	1865	1	0.8% (0.1–7.8)	4.04 (0.47–34.9)	0.20	3.77 (0.44–32.7)	0.23
Benign indication^5^	541	9824	22	6.6% (4.1–10.6)	579	10,265	10	3.0% (1.5–5.9)	2.30 (1.12–4.72)	0.02	2.14 (1.03–4.43)	0.04
No indication^6^	795	14,380	11	2.4% (1.2–4.9)	856	15,416	5	0.9% (0.4–2.2)	2.34 (0.87–6.30)	0.09	2.30 (0.83–6.35)	0.11
Ever smokers	584	10,371	32	9.3% (6.2–13.9)	582	10,520	15	3.9% (2.3–6.9)	2.22 (1.24–3.97)	0.007	2.17 (1.21–3.92)	0.01
Never smokers	752	13,833	1	0.1% (0.0–1.1)	853	15,161	0	0.0% (0.0–0.0)	—	—	—	—
BMI ≥ 30 kg/m^2^	438	7641	9	2.5% (1.3–4.8)	367	6563	5	2.4% (0.8–7.3)	1.54 (0.60–3.93)	0.37	2.13 (0.81–5.59)	0.12
BMI < 30 kg/m^2^	898	16,563	24	4.7% (2.9–7.4)	1068	19,117	10	1.7% (0.9–3.1)	2.77 (1.32–5.80)	0.007	2.19 (1.04–4.64)	0.04
Chronic bronchitis	1336	23,391	69	5.9% (4.5–7.8)	1435	25,326	34	3.1% (2.3–4.3)	2.19 (1.47–3.27)	<0.001	1.77 (1.18–2.67)	0.006
Age ≤45 y	825	14,447	46	6.4% (4.6–8.8)	897	15,633	19	2.8% (1.9–4.3)	2.63 (1.55–4.47)	<0.001	2.15 (1.24–3.71)	0.006
Estrogen >49^4^	419	5363	4	0.9% (0.3–2.7)	398	5163	5	1.2% (0.4–3.2)	0.76 (0.20–2.84)	0.68	0.72 (0.16–3.26)	0.67
No estr. or ≤49	258	2502	6	1.5% (0.5–5.0)	252	2591	1	0.4% (0.0–3.6)	6.23 (0.75–51.8)	0.09	3.80 (0.42–34.3)	0.23
Age 46 to 49 y	511	8944	23	5.5% (3.3–9.1)	538	9693	15	3.8% (2.4–6.0)	1.65 (0.88–3.10)	0.12	1.31 (0.68–2.52)	0.42
Estrogen >49^4^	371	6216	15	6.4% (3.1–13.3)	379	6572	10	3.4% (1.9–6.1)	1.58 (0.71–3.52)	0.26	1.41 (0.62–3.19)	0.41
No estr. or ≤49	127	1704	4	2.1% (0.5–7.8)	127	1843	3	2.2% (0.6–8.6)	1.33 (0.31–5.71)	0.70	1.23 (0.28–5.30)	0.78
Benign indication^5^	541	9443	34	7.0% (4.7–10.3)	579	10,170	16	3.8% (2.4–6.0)	2.25 (1.26–4.02)	0.006	1.83 (1.01–3.32)	0.047
No indication^6^	795	13,948	35	5.1% (3.4–7.5)	856	15,156	18	2.9% (1.9–4.4)	2.11 (1.21–3.70)	0.009	1.57 (0.87–2.84)	0.13
Ever smokers	584	9982	41	9.2% (6.4–13.2)	582	10,372	19	3.9% (2.5–6.1)	2.14 (1.28–3.58)	0.004	2.09 (1.23–3.54)	0.006
Never smokers	752	13,409	28	3.9% (2.7–5.7)	853	14,954	15	2.5% (1.6–4.0)	2.22 (1.17–4.22)	0.01	1.60 (0.83–3.10)	0.16
BMI ≥ 30 kg/m^2^	438	7217	33	9.9% (6.5–15.1)	367	6359	18	5.8% (3.7–9.1)	1.64 (0.94–2.86)	0.08	1.49 (0.84–2.64)	0.17
BMI < 30 kg/m^2^	898	16,174	36	4.5% (3.1–6.5)	1068	18,967	16	2.0% (1.3–3.2)	2.61 (1.47–4.65)	0.001	2.21 (1.23–3.98)	0.008

*BMI* body mass index, *CI* confidence interval.

^1^Hazard ratios were calculated using Cox proportional hazards models with age as the time scale.

^2^Hazard ratios were calculated using Cox proportional hazards models with age as the time scale and including inverse probability weights derived from a logistic regression model including 16 chronic conditions present at baseline, years of education (≤12, 13–16, >16), quartiles of household income (<$42,000, $42,000–56,999, $57,000–71,999, ≥$72,000), race (white vs. nonwhite), BMI (≥30 kg/m^2^ vs. <30), cigarette smoking (current or former vs. never), age at index date (continuous), and calendar year at index date (continuous). These weights were calculated separately in each stratum to maximize the balance at index date. No significant interactions were found by age, estrogen therapy, indication, smoking status, or by BMI for any of the two outcomes.

^3^Absolute cumulative risk at 25 years after bilateral oophorectomy (or index date) calculated using the Kaplan-Meier method and including inverse probability weights derived from a logistic regression model. These weights were calculated separately in each stratum to maximize the balance at index date.

^4^Women who were taking systemic estrogen therapy (only oral or transdermal) on their 50th birth date, after bilateral oophorectomy. Women who died or were lost to follow-up prior to their 50th birth date, or had not reached age 50 years as of 31 December 2018 were not included in this analysis. Follow-up for these analyses was started at age 50 years.

^5^The benign condition (e.g., cysts, endometrioma) was listed by the gynecologist in the medical record at the time of oophorectomy, but may not have been the sole indication for the surgery.

^6^Women without a benign ovarian condition. Historically, the terms “prophylactic”, “elective”, or “incidental” oophorectomy were used; however, we prefer to avoid these terms.

**Fig. 2:**
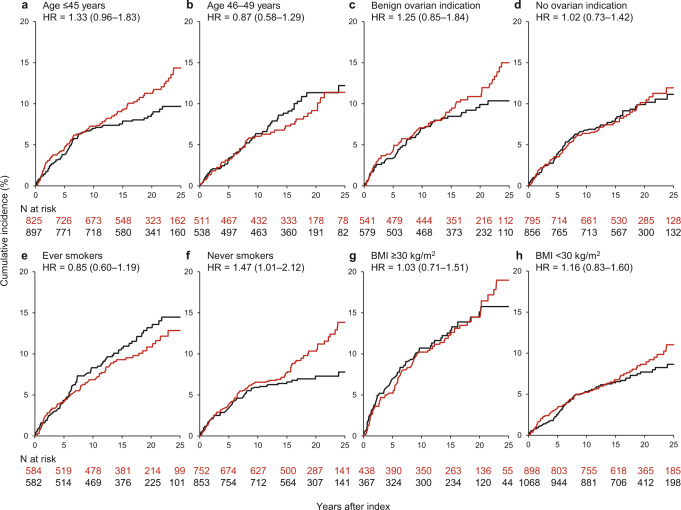
Cumulative incidence curves for all asthma in women who underwent bilateral oophorectomy compared with referent women. **a**, **b** Strata by age at oophorectomy; **c**, **d** strata by surgical indication; **e**, **f** strata by smoking status; **g**, **h** strata by body mass index at index date. The curves were weighted using inverse probability weights derived from a logistic regression model including 16 chronic conditions present at index, years of education, quartiles of household income, race, body mass index, cigarette smoking, and age and calendar year at index date (details provided in text). The interaction was significant by smoking status (*P* = 0.03), but not by age at oophorectomy, surgical indication, or by body mass index. *BMI* body mass index, *HR* hazard ratio.

**Fig. 3:**
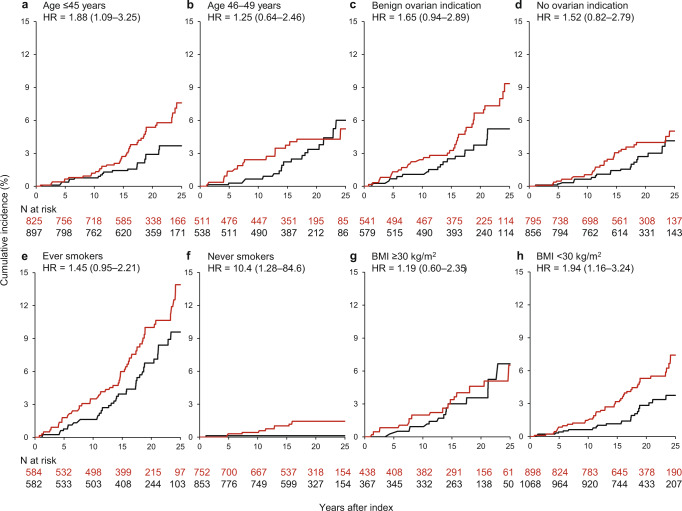
Cumulative incidence curves for all chronic obstructive pulmonary disease in women who underwent bilateral oophorectomy compared with referent women. **a**, **b** Strata by age at oophorectomy; **c**, **d** strata by surgical indication; **e**, **f** strata by smoking status; **g**, **h** strata by body mass index at index date. The curves were weighted using inverse probability weights derived from a logistic regression model including 16 chronic conditions present at index, years of education, quartiles of household income, race, body mass index, cigarette smoking, and age and calendar year at index date (details provided in text). The interactions were not significant by age at oophorectomy, surgical indication, smoking status, or by body mass index. *BMI* body mass index, *HR* hazard ratio.

The results for all asthma and all COPD outcomes after oophorectomy are shown in Table 3 and in Figs. 2 and 3. Although there was a stronger association of oophorectomy with all asthma for women who took estrogen therapy after oophorectomy in the ≤45 years age group, the interaction by age at oophorectomy was not significant. However, the interaction by smoking status was significant (*P* = 0.03), with an increased risk observed in never smokers who underwent oophorectomy compared to never smokers in the referent cohort (HR, 1.47; 95% CI, 1.01–2.12; ARI, 6.0%). There was no significant association in the smoking stratum (Fig. 2). The association of oophorectomy with all COPD was stronger for women aged ≤45 years at oophorectomy (HR, 1.88; 95% CI, 1.09–3.25; 95% CI, ARI, 4.4%), for never smokers (HR, 10.4; 95% CI, 1.28–84.6; ARI, 1.9%), and for non-obese women (HR, 1.94; 95% CI, 1.16–3.24; ARI, 3.6%); however, there were no significant interactions for any of the stratified analyses (Fig. 3).

The results for confirmed asthma and confirmed COPD outcomes after oophorectomy are shown in Supplementary Table 4. There was no association of oophorectomy with confirmed asthma, but there was an association with confirmed COPD among never smokers (HR, 9.45, 95% CI, 1.14–78.2, ARI, 1.7%), and the interaction by smoking status was significant (*P* = 0.04).

The results for emphysema and chronic bronchitis outcomes after oophorectomy are shown in Table 4. Within the smoking stratum there was a stronger association of oophorectomy with emphysema (HR, 2.17, 95% CI, 1.21–3.92, ARI, 5.4%) and with chronic bronchitis (HR, 2.09, 95% CI, 1.23–3.54, ARI, 5.3%). Non-obese women had a stronger association of oophorectomy with emphysema (HR, 2.19; 95% CI, 1.04–4.64; ARI, 3.0%) and with chronic bronchitis (HR, 2.21; 95% CI, 1.23–3.98; ARI, 2.5%). However, the interactions by smoking or obesity were not significant (Table 4).

### Sensitivity analyses

In the first set of sensitivity analyses, referent women who underwent bilateral oophorectomy after index date were censored at the date of oophorectomy. In the second set of sensitivity analyses, we considered the risk of *de novo* OLD within the subset of women with none of the 16 chronic conditions at index date (702 women who underwent oophorectomy and 925 referent women). The results from both sets of sensitivity analyses were virtually the same as the results from the primary analyses (data not shown).

## Discussion

**Fig. 4:**
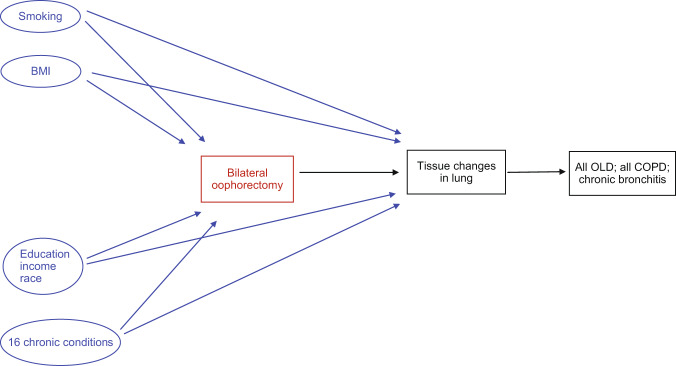
Causal diagram. Possible confounding effect of the variables included in the inverse probability weighting. Smoking and obesity were also considered as effect modifiers and stratified analyses were reported. *BMI* body mass index, *COPD* chronic obstructive pulmonary disease, *OLD* obstructive lung disease.

In this population-based cohort study, we evaluated the impact of bilateral oophorectomy on the risk of OLD. We found an overall higher risk of all OLD, all COPD, emphysema, and chronic bronchitis. The increased risk of all OLD was not explained by smoking status or by obesity. Indeed, there was a stronger association of oophorectomy with all OLD in never smokers and in non-obese women. We suggest that smoking and obesity may be stronger risk factors for OLD than oophorectomy. Therefore, the effect of oophorectomy becomes detectable only in women without these stronger risk factors. An increased risk for asthma was observed only in women ≤45 years who took estrogen therapy after oophorectomy and in never smokers. This study highlights that patients with oophorectomy do have increased respiratory symptoms associated with a clinical diagnosis of OLD. Figure 4 shows a general causal diagram including the possible effects of smoking, obesity, and other confounders or modifiers considered in our analyses.

A previous study using the same MOA-2 cohorts used in this study showed an increased rate of accumulation of several chronic conditions after oophorectomy, including asthma and COPD. However, asthma and COPD were defined only using diagnostic codes^27^. In the current study, we did not observe an increased risk of asthma or COPD when the diagnosis required confirmation by PFT. In clinical practice, patients are frequently diagnosed with COPD or asthma without PFT data. The diagnosis of asthma is primarily based on clinical history, and pulmonary function testing can be normal in asthmatics who have mild disease or are well treated at the time of testing. Based on GOLD guidelines, COPD should be diagnosed based on persistent post-bronchodilator obstruction. In our study, we did not find an association with COPD confirmed by PFT data; however, we did observe an increase in chronic bronchitis and emphysema which do not require PFT data for diagnosis.

Several prior studies investigated the effects of sex on respiratory disease by studying the relationship between menopause and lung function or COPD. Using the UK Biobank, Amaral et al. reported that women who underwent spontaneous menopause, especially at early ages, experienced a lower FVC, FEV_1_, but not FEV_1_/FVC ratio – suggesting a more restrictive than obstructive process in menopause. This association was stronger in women with earlier spontaneous menopause and in women who underwent surgical menopause^26^. Tang et al. also used the UK biobank data with similar results and noted that early menopause was associated with higher risk of COPD-related hospitalization and death. They also noted that contraceptive use was associated with lower risk of COPD-related hospitalization and death, and with higher FEV_1_ and FVC^37^. In our study, we found an increased risk of overall OLD which included COPD, chronic bronchitis, and emphysema in women who underwent bilateral oophorectomy. However, we did not see a clear association with COPD when applying stringent GOLD criteria, probably because many of the patients did not have pulmonary function data. COPD, chronic bronchitis, and emphysema are frequently diagnosed without PFT in medical practice, particularly in primary care and community settings. Our study highlights real world associations between oophorectomy and OLD.

There is less evidence in the literature relating asthma to menopause, especially to menopause induced by oophorectomy. The risk of severe asthma decreases in women after the age of 45 years, but not in men^38^; this decrease has been explained by the change in hormones associated with menopause. The Nurses’ Health Study and other studies suggested that spontaneous menopause is protective against asthma, but the protection is reversed by estrogen therapy^20,24,39^. Troisi et al. reported that the risk of asthma was associated with duration of estrogen therapy in both current and past users^20^. Jarvis et al. reported an association of estrogen therapy with asthma, particularly in non-obese women. They suggested that lean women have less circulating endogenous estrogen and are more responsive to exogenous estrogen^21^. However, in these studies, information about menopause, estrogen therapy, and asthma was collected via interview and may be incomplete or biased. By contrast, our study used medical records information to confirm the association of estrogen therapy with increased risk of asthma in women who underwent oophorectomy.

In the current study, the risk of asthma after oophorectomy was increased among never smokers and in women less than 46 years of age who received estrogen therapy. However, our findings may not be directly comparable with findings from previous studies because spontaneous menopause and oophorectomy are not equivalent processes. In spontaneous menopause, the reduction of hormonal levels is slow and progressive, whereas in oophorectomy there is an abrupt reduction that may affect airway and lung biology differently. Matulonga-Diakiese et al. suggested that the increased risk of asthma after surgical menopause may be due to the loss of testosterone that has an immunomodulatory effect against asthma^23^.

Obesity may modify the association between oophorectomy and different types of OLD. In our study, the association was stronger in non-obese women for all OLD, all COPD, and chronic bronchitis, but was similar for emphysema and asthma (non-significant differences). By contrast, Matulonga-Diakiese et al. reported an association of spontaneous menopause with increased risk of asthma restricted to obese women and explained the association with the higher levels of circulating estrogen in obese women^23^.

There are several strengths to our study. First, to the best of our knowledge, this is the first large, population-based cohort study to compare the effects of oophorectomy on the development of OLD to a referent group. Second, we had direct access to medical record documentation of the oophorectomies. Third, we validated OLD diagnoses using spirometric measurements including post-bronchodilator and bronchoprovocation data, and we applied current GOLD and GINA guidelines. Finally, we balanced our cohorts for possible confounders including age at index date, BMI, race, education, smoking history, 16 chronic conditions, and others.

There are also several limitations to our study. First, our study involved a single geographical location in the United States, and the observed findings may differ in other populations. Second, using the strict GOLD and GINA criteria to confirm COPD and asthma may have caused some misclassification. For instance, using an FEV_1_/FVC ratio of <70% may misclassify obstruction in older patients^40^. In addition, the devices used for spirometry were not standardized, and may have varied across care providers and over time.

Third, a large proportion of women in both cohorts developed “possible asthma”. These women were clinically diagnosed with asthma without documented PFT. However, when we applied the stringent GINA criteria, there was no increased risk of asthma in women with oophorectomy. The discrepancies in diagnosis of OLD are not surprising. Discordance of asthma and COPD diagnosis has been well described^41–44^. One study reported that only 55% of doctor-diagnosed asthma and 56% of doctor-diagnosed COPD had spirometry performed. Among patients with spirometry, 70% of asthma patients and 10% of COPD patients had concordant spirometry patterns^41^. Other studies suggested that ~70% of COPD worldwide may be underdiagnosed, whereas up to 60% may be overdiagnosed. Approximately 46% of these overdiagnosed patients are receiving respiratory medications^42,43^. Moreover, women are more likely to be underdiagnosed for OLD than men^45–47^.

There is mounting evidence that sex hormones play a role in the development and disease severity of OLD. Women who underwent bilateral oophorectomy in a population-based cohort study had a higher risk of asthma primarily if they underwent oophorectomy at a younger age and received estrogen therapy after the oophorectomy. Therefore, our findings suggest that the age of onset of menopause and estrogen therapy may influence the risk of asthma. They also suggest that the timing of hormone cessation and hormone replacement may play a role in lung pathology. We also found an association of oophorectomy with the development of COPD, chronic bronchitis, and emphysema. Although a clear sexual dimorphism in airway pathology exists, further investigation comparing spontaneous and surgical menopause is warranted to better understand the impact of hormonal changes on the lung throughout the lifespan of women.

The results of this study, combined with the increased risk of several chronic diseases and of multimorbidity reported from previous studies, suggest that bilateral oophorectomy for benign indications in premenopausal women should be avoided unless there is clear evidence of a high genetic risk of ovarian cancer^27^. The harmful effect of bilateral oophorectomy on the lung, heart, brain, bone, and other organs and systems outweigh the prevention of ovarian cancer in women at average risk.

## Supplementary information

REPORTING SUMMARY

Supplementary Information

## Data Availability

The tables of results from the sensitivity analyses are available from the corresponding author upon request. By contrast, to obtain the data that support the findings of this study, the user must submit a brief outline of the intended use of the data (no longer than a page). In addition, the user will be required to sign a data-sharing agreement, and provide payment to the Mayo Clinic for administrative costs in the preparation of the shared file of de-identified data. The application for data can be submitted with a brief outline of the data need to the corresponding author Megan Dulohery Scrodin.
