# Supplementary material for: A population-based cohort study on the risk of obstructive lung disease after bilateral oophorectomy
Source: NPJ Prim Care Respir Med. 2022 Nov 15;32:52. doi: 10.1038/s41533-022-00317-4 (PMC9663719; doi:10.1038/s41533-022-00317-4)
Supplement: Supplementary file 1 — REPORTING SUMMARY [file 41533_2022_317_MOESM1_ESM.pdf]

nature portfolio

Corresponding author(s): Megan M. Dulohery Scrodin

Last updated by author(s): Oct 5, 2022

Reporting Summary

Nature Portfolio wishes to improve the reproducibility of the work that we publish. This form provides structure for consistency and transparency in reporting. For further information on Nature Portfolio policies, see our Editorial Policies and the Editorial Policy Checklist.

Statistics

For all statistical analyses, confirm that the following items are present in the figure legend, table legend, main text, or Methods section.

n/a	Confirmed	
☐	☒	The exact sample size (*n*) for each experimental group/condition, given as a discrete number and unit of measurement
☐	☒	A statement on whether measurements were taken from distinct samples or whether the same sample was measured repeatedly
☐	☒	The statistical test(s) used AND whether they are one- or two-sided<br>*Only common tests should be described solely by name; describe more complex techniques in the Methods section.*
☐	☒	A description of all covariates tested
☐	☒	A description of any assumptions or corrections, such as tests of normality and adjustment for multiple comparisons
☐	☒	A full description of the statistical parameters including central tendency (e.g. means) or other basic estimates (e.g. regression coefficient) AND variation (e.g. standard deviation) or associated estimates of uncertainty (e.g. confidence intervals)
☐	☒	For null hypothesis testing, the test statistic (e.g. *F*, *t*, *r*) with confidence intervals, effect sizes, degrees of freedom and *P* value noted<br>*Give P values as exact values whenever suitable.*
☒	☐	For Bayesian analysis, information on the choice of priors and Markov chain Monte Carlo settings
☒	☐	For hierarchical and complex designs, identification of the appropriate level for tests and full reporting of outcomes
☒	☐	Estimates of effect sizes (e.g. Cohen's *d*, Pearson's *r*), indicating how they were calculated

Our web collection on statistics for biologists contains articles on many of the points above.

Software and code

Policy information about availability of computer code

Data collection	No software was used for data collection.
Data analysis	Analyses were performed using SAS version 9.4 (SAS Institute Inc, Cary, NC).

For manuscripts utilizing custom algorithms or software that are central to the research but not yet described in published literature, software must be made available to editors and reviewers. We strongly encourage code deposition in a community repository (e.g. GitHub). See the Nature Portfolio guidelines for submitting code & software for further information.

Data

Policy information about availability of data

All manuscripts must include a data availability statement. This statement should provide the following information, where applicable:

- Accession codes, unique identifiers, or web links for publicly available datasets
- A description of any restrictions on data availability
- For clinical datasets or third party data, please ensure that the statement adheres to our policy

The tables of results from the sensitivity analyses are available from the corresponding author. The data that support the findings of this study can be obtained from the corresponding author upon request. The request should include a brief outline of the intended use of the data (no longer than a page). In addition, sharing of de-identified data will require a data sharing agreement and a payment for the preparation of the shared file.

March 2021

Human research participants

Policy information about studies involving human research participants and Sex and Gender in Research.

Reporting on sex and gender	All subjects in the study were women. We used the term "sex" as a biological attribute because women were not interviewed, and gender was not collected. Sex was derived from medical record information.
Population characteristics	The population characteristics abstracted from the medical record were reported in Table 2.
Recruitment	This study was population-based and we have no concern about selection bias.
Ethics oversight	Women were not contacted directly as part of the study and were excluded if they did not authorize the use of their medical record for research (only 2.5% of women in the overall population). Research activities were approved by both the Mayo Clinic and the Olmsted Medical Center Institutional Review Boards.

Note that full information on the approval of the study protocol must also be provided in the manuscript.

Field-specific reporting

Please select the one below that is the best fit for your research. If you are not sure, read the appropriate sections before making your selection.

☒ Life sciences ☐ Behavioural & social sciences ☐ Ecological, evolutionary & environmental sciences

For a reference copy of the document with all sections, see nature.com/documents/nr-reporting-summary-flat.pdf

Life sciences study design

All studies must disclose on these points even when the disclosure is negative.

Sample size	This was a population-based study, including all eligible women in the sample. This was a complete enumeration of all women who underwent bilateral oophorectomy in Olmsted County, and the same number of age-matched referent women.
Data exclusions	Women were not contacted directly as part of the study and were excluded if they did not authorize the use of their medical record for research (only 2.5% of women in the overall population).
Replication	This is an observational study, with complete enumeration of the population. Replication was not possible.
Randomization	This study did not involve randomization. The covariates were controlled using inverse probability weighting to balance the two cohorts on baseline characteristics.
Blinding	Assessment of lung disease characteristics was conducted by investigators who were kept unaware of the oophorectomy status.

Reporting for specific materials, systems and methods

We require information from authors about some types of materials, experimental systems and methods used in many studies. Here, indicate whether each material, system or method listed is relevant to your study. If you are not sure if a list item applies to your research, read the appropriate section before selecting a response.

Materials & experimental systems

n/a	Involved in the study	
☒	☐	Antibodies
☒	☐	Eukaryotic cell lines
☒	☐	Palaeontology and archaeology
☒	☐	Animals and other organisms
☒	☐	Clinical data
☒	☐	Dual use research of concern

Methods

n/a	Involved in the study	
☒	☐	ChIP-seq
☒	☐	Flow cytometry
☒	☐	MRI-based neuroimaging
