# Supplementary material for: A population-based cohort study on the risk of obstructive lung disease after bilateral oophorectomy
Source: NPJ Prim Care Respir Med. 2022 Nov 15;32:52. doi: 10.1038/s41533-022-00317-4 (PMC9663719; doi:10.1038/s41533-022-00317-4)
Supplement: Supplementary file 2 — Supplementary Information [file 41533_2022_317_MOESM2_ESM.docx]

**Supplementary information**

**A population-based cohort study on the risk of obstructive lung disease after bilateral oophorectomy**

Trinh T. Nguyen, DO, Carin Y. Smith, BS, Liliana Gazzuola Rocca, MD, Walter A. Rocca, MD, MPH, Robert Vassallo, MD, Megan M. Dulohery Scrodin, MD

**Supplementary Table 1.** Diagnostic codes used to screen women for obstructive lung disease.

| **DHHS Category** | **OLD Type** | **Diagnostic Code** | **Code Type** | **Description** |
| --- | --- | --- | --- | --- |
| Asthma | Asthma | 493.xx | ICD-9 | Asthma |
|  |  | J45.xxx | ICD-10 | Asthma |
| COPD | COPD | 494.x | ICD-9 | Bronchiectasis |
|  |  | 496 | ICD-9 | Chronic airway obstruction, not elsewhere classified |
|  |  | J44.x | ICD-10 | Other chronic obstructive pulmonary disease |
|  |  | J47.x | ICD-10 | Bronchiectasis |
|  | Emphysema | 492.xx | ICD-9 | Emphysema |
|  |  | J43.x | ICD-10 | Emphysema |
|  | Chronic bronchitis | 490 | ICD-9 | Bronchitis, not specified as acute or chronic |
|  |  | 491.xx | ICD-9 | Chronic bronchitis |
|  |  | J40 | ICD-10 | Bronchitis, not specified as acute or chronic |
|  |  | J41.x | ICD-10 | Simple and mucopurulent chronic bronchitis |
|  |  | J42 | ICD-10 | Unspecified chronic bronchitis |
| *COPD* chronic obstructive pulmonary disease, *DHHS* Department of Health and Human Services, *ICD* International Classification of Diseases. | | | | |

**Supplementary Table 2.** Follow-up and menopausal characteristics of women who underwent bilateral oophorectomy and referent women.

| **Characteristic** | **Bilateral oophorectomy** | **Referent women** | ***P* value** |
| --- | --- | --- | --- |
| **All women, *n*** | **1336** | **1435** |  |
| Age at end of follow-up (y), median (IQR) | 62.0 (57.0-67.0) | 62.0 (57.0-67.0) | 0.36 |
| Length of follow-up (y), median (IQR) | 18.7 (14.2-23.6) | 18.5 (14.1-23.3) | 0.34 |
| Status at end of follow-up status, *n* (%) |  |  | 0.002 |
| Deceased | 88 (6.6%) | 59 (4.1%) |  |
| Lost to follow-up | 256 (19.2%) | 328 (22.9%) |  |
| Alive | 992 (74.3%) | 1048 (73.0%) |  |
| Menopause status, *n* (%) |  |  | -- |
| No | -- | 181 (12.7%) |  |
| Yes | 1336 (100.0%) | 1244 (87.3%) |  |
| Unknown | -- | 10 |  |
|  |  |  |  |
| **Women who underwent menopause, *n*** | **1336** | **1244** |  |
| Age at menopause (y), median (IQR) | 44.0 (40.0-47.0) | 50.0 (46.5-52.0) | <0.001 |
| Menopause timing, *n* (%) |  |  | -- |
| Before index | 121 (9.1%) | 206 (16.6%) |  |
| Concurrent with index | 1215 (90.9%) | -- |  |
| After index | -- | 1032 (83.4%) |  |
| Unknown | -- | 6 |  |
| Menopause type, *n* (%) |  |  | -- |
| Spontaneous | -- | 951 (76.5%) |  |
| Surgical | 1336 (100.0%) | 278 (22.4%) |  |
| Hysterectomy or thermal ablation | 121 (9.1%) | 186 (15.0%) |  |
| Hysterectomy and bilateral oophorectomy | 1198 (89.7%) | 85 (6.8%) |  |
| Bilateral oophorectomy | 17 (1.3%) | 7 (0.6%) |  |
| Chemical or radiation | -- | 14 (1.1%) |  |
| Unknown | -- | 1 |  |
| Systemic estrogen therapy, *n* (%) |  |  | <0.001 |
| No | 121 (9.1%) | 791 (64.4%) |  |
| Yes | 1213 (90.9%) | 438 (35.6%) |  |
| Unknown | 2 | 15 |  |
|  |  |  |  |
| **Women with estrogen therapy, *n*** | **1213** | **438** |  |
| Age at end of estrogen therapy (y), median (IQR) | 52.9 (48.6-57.9) | 51.7 (47.7-56.4) | 0.005 |
| Length of estrogen therapy (y), median (IQR) | 8.8 (4.4-14.1) | 5.9 (2.9-10.1) | <0.001 |
| Earliest estrogen therapy type, *n* (%) |  |  | <0.001 |
| Conjugated estrogen | 850 (70.1%) | 289 (66.0%) |  |
| Estradiol | 335 (27.6%) | 120 (27.4%) |  |
| Other^1^ | 27 (2.2%) | 29 (2.0%) |  |
| Unknown | 1 | 0 |  |

**Supplementary Table 2.** Continued.

| **Characteristic** | **Bilateral oophorectomy** | **Referent women** | ***P* value** |
| --- | --- | --- | --- |
| Earliest estrogen therapy route, *n* (%) |  |  | <0.001 |
| Oral | 945 (78.0%) | 355 (81.1%) |  |
| Patch | 267 (22.0%) | 79 (18.0%) |  |
| Other^2^ | 0 (0.0%) | 4 (0.9%) |  |
| Unknown | 1 | 0 |  |
| ^1^ Other estrogen therapy types include esterified estrogen, estropipate, and ethinyl estradiol.  ^2^ Other estrogen therapy routes include parenteral, vaginal ring, or systemic transdermal gel. | | | |

**Supplementary Table 3.** Clinical characteristics of women with de novo asthma or COPD outcomes.

| **Characteristic** |  | **All asthma** | |  |  | **All COPD** | |  |
| --- | --- | --- | --- | --- | --- | --- | --- | --- |
|  | **Bilateral oophorectomy**  **(*n* = 133)** | | **Referent women**  **(*n* = 121)** | | **Bilateral oophorectomy**  **(*n* = 59)** | | **Referent women**  **(*n* = 37)** | |
| Age at index date (y), median (IQR) | 44.0 (40.0-47.0) | | 44.0 (40.0-47.0) | | 42.0 (39.0-47.0) | | 44.0 (42.0-48.0) | |
| Age at OLD diagnosis (y), median (IQR) | 51.0 (45.0-56.0) | | 50.0 (45.0-56.0) | | 54.0 (52.0-59.0) | | 60.0 (53.0-63.0) | |
| BMI at index date (kg/m^2^), median (IQR) | 28.3 (23.6-34.9) | | 29.2 (24.3-34.6) | | 25.0 (22.2-30.9) | | 28.5 (24.3-32.9) | |
| Smoking status, *n* (%) |  | |  | |  | |  | |
| Never | 74 (55.6%) | | 53 (43.8%) | | 8 (13.6%) | | 1 (2.7%) | |
| Former | 30 (22.6%) | | 31 (25.6%) | | 5 (8.5%) | | 6 (16.2%) | |
| Current | 29 (21.8%) | | 37 (30.6%) | | 46 (78.0%) | | 30 (81.1%) | |
| Smoking pack-years, *n* (%) |  | |  | |  | |  | |
| 0 | 74 (55.6%) | | 53 (43.8%) | | 8 (13.6%) | | 1 (2.7%) | |
| 0.1-5.9 | 14 (10.5%) | | 13 (10.7%) | | 2 (3.4%) | | 2 (5.4%) | |
| ≥6.0 | 45 (33.8%) | | 53 (43.8%) | | 48 (81.4%) | | 34 (91.9%) | |
| Any chest CT, *n* (%) | 68 (51.1%) | | 52 (43.0%) | | 53 (89.8%) | | 35 (94.6%) | |
| Number of chest CT, median (IQR) | 2.0 (1.0-4.0) | | 2.0 (1.0-5.0) | | 4.0 (2.0-6.0) | | 3.0 (2.0-5.0) | |
| Any abnormal chest CT, *n* (%) | 54 (40.6%) | | 48 (39.7%) | | 53 (89.8%) | | 35 (94.6%) | |
| Number of abnormal chest CT, median (IQR) | 2.0 (1.0-4.0) | | 1.5 (1.0-4.0) | | 4.0 (2.0-6.0) | | 2.0 (1.0-5.0) | |
| Any PFT, *n* (%) | 80 (60.2%) | | 68 (56.2%) | | 50 (84.7%) | | 31 (83.8%) | |
| Number of PFT, median (IQR) | 2.0 (1.0-4.0) | | 2.0 (1.0-4.0) | | 2.5 (1.0-4.0) | | 1.0 (1.0-4.0) | |
| Any abnormal PFT, *n* (%) | 51 (38.3%) | | 42 (34.7%) | | 27 (45.8%) | | 21 (56.8%) | |
| Number of abnormal PFT, median (IQR) | 1.0 (1.0-2.0) | | 1.0 (1.0-3.0) | | 2.0 (1.0-3.0) | | 1.0 (1.0-3.0) | |
| Any hospital visits for exacerbation, *n* (%)^1^ | 1 (0.8%) | | 1 (0.8%) | | 1 (1.7%) | | 1 (2.7%) | |
| Any treatment at OLD diagnosis, *n* (%) | 88 (66.2%) | | 73 (60.3%) | | 25 (42.4%) | | 17 (45.9%) | |
| Any treatment at end of follow-up, *n* (%) | 84 (63.2%) | | 71 (58.7%) | | 33 (55.9%) | | 24 (64.9%) | |
| Confirmed OLD, *n* (%) | 46 (34.6%) | | 35 (28.9%) | | 22 (37.3%) | | 18 (48.6%) | |
| FEV_1_, median (IQR)^2^ | 1.8 (1.6-2.5) | | 1.8 (1.5-2.3) | | 1.7 (1.5-2.2) | | 1.2 (1.0-1.6) | |
| FVC, median (IQR)^2^ | 2.8 (2.3-3.2) | | 2.6 (2.3-3.2) | | 2.9 (2.5-3.3) | | 2.3 (1.7-3.0) | |
| FEV_1_/FVC, median (IQR)^2^ | 68.8 (59.7-78.0) | | 67.6 (64.6-76.2) | | 64.9 (59.7-67.8) | | 63.7 (49.8-64.4) | |
| Bronchodilation, *n* (%)^2^ | 40 (87.0%) | | 31 (88.6%) | | 22 (100.0%) | | 18 (100.0%) | |
| FEV_1_, median (IQR)^2^ | 2.0 (1.6-2.4) | | 1.8 (1.6-2.4) | | 1.9 (1.7-2.2) | | 1.4 (1.0-1.7) | |
| FEV_1_/FVC, median (IQR)^2^ | 71.8 (63.8-79.3) | | 72.2 (66.9-76.6) | | 68.0 (61.4-69.4) | | 64.7 (51.6-68.5) | |
| Bronchoprovocation, *n* (%)^2^ | 22 (47.8%) | | 18 (51.4%) | | -- | | -- | |
| FEV_1_, median (IQR)^2^ | 1.8 (1.7-2.1) | | 1.6 (1.4-1.9) | | -- | | -- | |
| FEV_1_/FVC, median (IQR)^2^ | 73.1 (68.7-80.1) | | 70.9 (66.1-74.0) | | -- | | -- | |
| *BMI* body mass index, *COPD* chronic obstructive pulmonary disease, *CT* computed tomography, *FEV_1_* forced expiratory volume at 1 second, *FVC* forced vital capacity, *IQR* interquartile range, *OLD* obstructive lung disease, *PFT* pulmonary function test.  ^1^ Includes hospital admissions and emergency department visits for exacerbation of asthma or COPD.  ^2^ Distributions were limited to women with obstructive PFT for confirmed asthma or confirmed COPD, with measurements from the earliest obstructive PFT available. | | | | | | | | |

**Supplementary Table 4.** Cumulative incidence of confirmed asthma and confirmed COPD overall and in strata by age at oophorectomy, estrogen therapy, surgical indication, cigarette smoking, and body mass index.

|  |  | **Bilateral oophorectomy** | | | |  |  | **Referent women** | | | |  |  | **Unweighted models^1^** | |  |  | **Weighted models^2^** | |  |
| --- | --- | --- | --- | --- | --- | --- | --- | --- | --- | --- | --- | --- | --- | --- | --- | --- | --- | --- | --- | --- |
| **Strata** | ***N* at risk** | | **Person-years** | ***N* of events** | **Absolute risk^3^ (95% CI)** | | ***N* at risk** | | **Person -years** | ***N* of events** | **Absolute risk^3^ (95% CI)** | | **Hazard ratio**  **(95% CI)** | | ***P* value** | | **Hazard ratio**  **(95% CI)** | | ***P* value** | |
| Confirmed asthma | 1336 | | 23,796 | 46 | 4.5% (3.2-6.1) | | 1435 | | 25,324 | 35 | 3.4% (2.4-4.7) | | 1.40 (0.92-2.13) | | 0.12 | | 1.24 (0.80-1.90) | | 0.33 | |
| Age ≤45 y | 825 | | 14,779 | 28 | 4.3% (2.8-6.4) | | 897 | | 15,658 | 16 | 2.6% (1.6-4.3) | | 1.86 (1.02-3.39) | | 0.04 | | 1.61 (0.85-3.02) | | 0.14 | |
| Estrogen >49^4^ | 434 | | 5551 | 8 | 3.6% (1.5-8.3) | | 401 | | 5186 | 4 | 1.6% (0.5-5.1) | | 1.86 (0.56-6.17) | | 0.31 | | 2.28 (0.64-8.11) | | 0.20 | |
| No estr. or ≤49 | 261 | | 2523 | 5 | 1.3% (0.4-4.7) | | 250 | | 2574 | 2 | 0.7% (0.1-3.6) | | 2.53 (0.49-13.1) | | 0.27 | | 1.73 (0.31-9.58) | | 0.53 | |
| Age 46 to 49 y | 511 | | 9017 | 18 | 4.8% (2.9-7.9) | | 538 | | 9666 | 19 | 4.7% (3.0-7.3) | | 1.01 (0.56-1.83) | | 0.96 | | 0.90 (0.48-1.66) | | 0.73 | |
| Estrogen >49^4^ | 371 | | 6251 | 10 | 3.7% (1.9-7.1) | | 380 | | 6578 | 13 | 4.4% (2.6-7.4) | | 0.81 (0.37-1.78) | | 0.60 | | 0.73 (0.32-1.64) | | 0.45 | |
| No estr. or ≤49 | 127 | | 1746 | 4 | 6.6% (1.6-25.2) | | 127 | | 1808 | 4 | 2.7% (0.8-9.3) | | 1.03 (0.26-4.14) | | 0.97 | | 1.17 (0.28-4.85) | | 0.83 | |
| Benign indication^5^ | 541 | | 9668 | 20 | 4.0% (2.5-6.4) | | 579 | | 10,184 | 15 | 3.7% (2.2-6.1) | | 1.38 (0.74-2.60) | | 0.31 | | 1.14 (0.60-2.20) | | 0.69 | |
| No indication^6^ | 795 | | 14,128 | 26 | 4.8% (3.1-7.4) | | 856 | | 15,140 | 20 | 3.3% (2.1-5.0) | | 1.40 (0.80-2.48) | | 0.24 | | 1.22 (0.67-2.22) | | 0.51 | |
| Ever smokers | 584 | | 10,290 | 25 | 5.7% (3.8-8.6) | | 582 | | 10,370 | 23 | 5.3% (3.5-7.9) | | 1.10 (0.65-1.88) | | 0.72 | | 1.09 (0.63-1.87) | | 0.77 | |
| Never smokers | 752 | | 13,506 | 21 | 3.7% (2.3-5.9) | | 853 | | 14,954 | 12 | 1.7% (1.0-2.9) | | 2.04 (1.02-4.07) | | 0.04 | | 1.86 (0.91-3.79) | | 0.09 | |
| BMI ≥30 kg/m^2^ | 438 | | 7447 | 18 | 5.0% (3.0-8.3) | | 367 | | 6446 | 13 | 4.4% (2.5-7.7) | | 1.19 (0.63-2.26) | | 0.59 | | 1.12 (0.57-2.17) | | 0.75 | |
| BMI <30 kg/m^2^ | 898 | | 16,349 | 28 | 4.2% (2.8-6.3) | | 1068 | | 18,878 | 22 | 2.7% (1.8-4.2) | | 1.47 (0.85-2.54) | | 0.17 | | 1.41 (0.81-2.46) | | 0.22 | |
|  |  | |  |  |  | |  | |  |  |  | |  | |  | |  | |  | |
| Confirmed COPD | 1336 | | 24,176 | 22 | 2.5% (1.6-4.0) | | 1435 | | 25,630 | 18 | 2.3% (1.4-3.8) | | 1.29 (0.69-2.41) | | 0.42 | | 1.26 (0.67-2.36) | | 0.48 | |
| Age ≤45 y | 825 | | 15,062 | 14 | 2.7% (1.5-4.9) | | 897 | | 15,779 | 9 | 1.3% (0.7-2.6) | | 1.63 (0.70-3.76) | | 0.25 | | 1.67 (0.71-3.92) | | 0.24 | |
| Estrogen >49^4^ | 439 | | 5635 | 8 | 2.9% (1.2-7.1) | | 404 | | 5231 | 4 | 1.4% (0.5-4.1) | | 1.86 (0.56-6.19) | | 0.31 | | 1.98 (0.53-7.40) | | 0.31 | |
| No estr. or ≤49 | 268 | | 2623 | 4 | 0.5% (0.1-3.6) | | 252 | | 2606 | 1 | 0.4% (0.0-3.5) | | 3.96 (0.44-35.7) | | 0.22 | | 1.28 (0.08-20.5) | | 0.86 | |
| Age 46 to 49 y | 511 | | 9114 | 8 | 2.2% (1.1-4.2) | | 538 | | 9851 | 9 | 3.7% (1.8-7.6) | | 0.96 (0.37-2.48) | | 0.93 | | 1.08 (0.40-2.90) | | 0.88 | |
| Estrogen >49^4^ | 374 | | 6333 | 7 | 2.5% (1.2-5.1) | | 381 | | 6698 | 7 | 4.2% (1.8-9.9) | | 1.05 (0.37-3.00) | | 0.92 | | 1.11 (0.35-3.53) | | 0.86 | |
| No estr. or ≤49 | 127 | | 1757 | 0 | 0.0% (0.0-0.0) | | 127 | | 1875 | 1 | 6.0% (0.9-34.7) | | -- | | -- | | -- | | -- | |
| Benign indication^5^ | 541 | | 9881 | 9 | 2.4% (1.2-4.7) | | 579 | | 10,264 | 8 | 2.0% (1.0-3.9) | | 1.17 (0.45-3.05) | | 0.74 | | 1.11 (0.42-2.91) | | 0.84 | |
| No indication^6^ | 795 | | 14,295 | 13 | 2.8% (1.5-5.3) | | 856 | | 15,366 | 10 | 2.7% (1.3-5.3) | | 1.38 (0.61-3.15) | | 0.44 | | 1.41 (0.61-3.25) | | 0.43 | |

**Supplementary Table 4.** Continued.

|  |  | **Bilateral oophorectomy** | | | |  |  | **Referent women** | | | |  |  | **Unweighted models^1^** | |  |  | **Weighted models^2^** | |  |
| --- | --- | --- | --- | --- | --- | --- | --- | --- | --- | --- | --- | --- | --- | --- | --- | --- | --- | --- | --- | --- |
| **Strata** | ***N* at risk** | | **Person-years** | ***N* of events** | **Absolute risk^3^ (95% CI)** | | ***N* at risk** | | **Person -years** | ***N* of events** | **Absolute risk^3^ (95% CI)** | | **Hazard ratio**  **(95% CI)** | | ***P* value** | | **Hazard ratio**  **(95% CI)** | | ***P* value** | |
| Ever smokers | 584 | | 10,412 | 15 | 3.6% (2.1-6.1) | | 582 | | 10,496 | 17 | 4.8% (2.8-8.1) | | 0.91 (0.46-1.81) | | 0.80 | | 0.91 (0.45-1.83) | | 0.78 | |
| Never smokers | 752 | | 13,764 | 7 | 1.8% (0.8-4.1) | | 853 | | 15,134 | 1 | 0.1% (0.0-0.9) | | 7.43 (0.94-58.7) | | 0.06 | | 9.45 (1.14-78.2) | | 0.04 | |
| BMI ≥30 kg/m^2^ | 438 | | 7647 | 6 | 3.3% (1.2, 9.0) | | 367 | | 6556 | 6 | 3.2% (1.2, 8.6) | | 0.84 (0.27-2.59) | | 0.76 | | 0.82 (0.26-2.58) | | 0.74 | |
| BMI <30 kg/m^2^ | 898 | | 16,529 | 16 | 2.4% (1.5, 3.9) | | 1068 | | 19,074 | 12 | 2.0% (1.1, 3.6) | | 1.55 (0.73-3.27) | | 0.25 | | 1.59 (0.75-3.37) | | 0.23 | |
| *BMI* body mass index, *CI* confidence interval, *COPD* chronic obstructive pulmonary disease.  ^1^ Hazard ratios were calculated using Cox proportional hazards models with age as the time scale.  ^2^ Hazard ratios were calculated using Cox proportional hazards models with age as the time scale and including inverse probability weights derived from a logistic regression model including 16 chronic conditions present at baseline, years of education (≤12, 13-16, >16), quartiles of household income (<$42,000, $42,000-56,999, $57,000-71,999, ≥$72,000), race (white vs. nonwhite), BMI (≥30 kg/m^2^ vs. <30), cigarette smoking (current or former vs. never), age at index date (continuous), and calendar year at index date (continuous). These weights were calculated separately in each stratum to maximize the balance at index date. A significant interaction was found by smoking for confirmed COPD (*P* = 0.04). No significant interactions were found by age, estrogen therapy, indication, or by BMI for any of the two outcomes.  ^3^ Absolute cumulative risk at 25 years after bilateral oophorectomy (or index date) calculated using the Kaplan-Meier method and including inverse probability weights derived from a logistic regression model. These weights were calculated separately in each stratum to maximize the balance at index date.  ^4^ Women who were taking systemic estrogen therapy (only oral or transdermal) on their 50^th^ birth date, after bilateral oophorectomy. Women who died or were lost to follow-up prior to their 50^th^ birth date, or had not reached age 50 years as of December 31, 2018 were not included in this analysis. Follow-up for these analyses was started at age 50 years.  ^5^ The benign condition (e.g., cysts, endometrioma) was listed by the gynecologist in the medical record at the time of oophorectomy, but may not have been the sole indication for the surgery.  ^6^ Women without a benign ovarian condition. Historically, the terms “prophylactic”, “elective”, or “incidental” oophorectomy were used; however, we prefer to avoid these terms. | | | | | | | | | | | | | | | | | | | | |

**Supplementary Figure 1**. **Flow chart of the two study cohorts.** Diagnostic codes for OLD were obtained electronically from the diagnostic indexes of the Rochester Epidemiology Project medical records-linkage system for all women in the bilateral oophorectomy and referent cohorts. Medical record review was used to determine OLD status, type of OLD, and diagnosis date for all women who met the screening criteria for OLD on or after index date (oophorectomy date).

*COPD* chronic obstructive pulmonary disease, *CT* computed tomography, *OLD* obstructive lung disease, *PFT* pulmonary function testing.

^1^ Screening criteria included one or more diagnostic codes for asthma, COPD, or emphysema, and two or more codes separated by >90 days and <1 year for chronic bronchitis**.**

**Supplementary Figure 2. Cumulative incidence curves for all obstructive lung disease in women who underwent bilateral oophorectomy compared with referent women.** Panels a and b, strata by age at oophorectomy; Panels c and d, strata by surgical indication; Panels e and f, strata by smoking status; and Panels g and h, strata by body mass index at index date. The curves were weighted using inverse probability weights derived from a logistic regression model including 16 chronic conditions present at index, years of education, quartiles of household income, race, body mass index, cigarette smoking, age at index date, and calendar year at index date. The interactions were not significant by age, surgical indication, smoking status, or by body mass index.

*BMI* body mass index, *HR* hazard ratio.
